# Hydroadipsia, and the Water Supply of Living Bodies, Clinically Illustrated*A term proposed to express absence of water thirst. Adipsia, absence of thirst; Hydro, water, Water thirst absent, lost, paralysed.

**Published:** 1876-06

**Authors:** Z. Collins McElroy

**Affiliations:** Zanesville, O.


### *HYDROADIPSIA, AND THE WATER SUPPLY OF LIVING BODIES, CLINICALLY ILLUSTRATED.

BY Z. COLLINS McELROY, M. D., ZANESVILLE, O.

Occupying no less than one-third of the entire surface of the world, present in variable quantities on the remainder; on the surface, as well as beneath it; in the atmosphere as vapor; ubiquitous and omnipresent in inorganic, and organic natures, constituting in weight an overwhelming proportion of all organic life; water, from in its organic constituents, no less than its volume, as well as the functions it performs, demands far more attention and consideration than it has ever received at the hands of medical men.

Its presence is an essential condition of organic life, animal and vegetable, from the simplest cell, to the most complex animal or vegetable existence. Nor does its functions stop here, for it is no less important in the realm of inorganic nature.

Nor are its boundaries even thus limited. Human industries, agriculture, manufactures, commerce, transportation, art, science, literature, everything connected with life in its individual, social and collective capacities, is more or less closely connected with, and dependent on water.

It is Nature's great solvent, taking up and holding in more or less limpid solutions the hardest rocks, and the most elastic gases; as well as most of the metals in certain chemical states. Ever passing in vapor upwards from the earth's surface into the atmosphere, even when falling condensed as rain drops, or as crystals in snow. The stream is ever upward and ever downward, varying from moment to moment as conditions vary, now as snow, again as rain, or dew, or mist.

No human body can exist without it, for either as water, or the elementary constituents of water, oxygen and hydro-

*A term proposed to express absence of water thirst. Adipsia, absence of thirst; Hydro, water, Water thirst absent, lost, paralysed.

gen, it forms nine-tenths of the total weight of it at all times during life.

There can be no blood, no cells, no liquids, no solids, not a single tissue, texture, or viscus can exist without it.

Can it be possible, or probable, that none of what we call pathological conditions of life are not connected with, or dependent on the proper supply and relations of water to other parts of living human bodies? It seems to me such an assumption would fall very short of representing the facts of life.

To illustrate that some human sickness is due to defective supply, or proportions of water, to individual life, allow me to place before you a brief history of a case about which I never was consulted, did not prescribe for, and yet was the unconscious instrumentality of relief to a very chronic sufferer.

Several years ago, while Col. John J. Douglas was postmaster in the city of Zanesville, he delivered to me a package of pamphlets, wrapper torn off, but held together by twine, extra copies of an article of mine published in a Medical Journal, illustrating the effects of water as a therapeutic agent, and asked me for a copy, which was readily given.

The incident quickly passed out of my mind, was forgotten, but I was destined to hear from it again in a very unexpected manner.

Last summer (1875), Col. Douglas called on me to tell me what had come of it. He became a sufferer from what he called an affection of the kidneys. Did not pass enough water from his bladder; and what he did pass was very highly colored, passed with more or less difficulty all the time, and sometimes with considerable uneasiness.

For this he consulted one or more physicians in this city, who prescribed for him, and he followed their instructions. Not finding any relief, he took some patent medicines, with no better success. Then he made a trip east, was examined and prescribed for in Philadelphia and New York, but with like results. He now ceased to take any medicines, having made up his mind that he could not get relief from them.

Two years since in looking over some pamphlets and papers, he came across the copy of the Medical Journal I had given him. In it he read my paper for the first time, having laid it aside at the time he got it, to read at a more convenient season, and concluded its suggestions applicable to himself, and carried them into effect, by simply drinking water, sometimes hot, made palatable with salt; at other times cold, but mostly cold, in much larger quantities than he had done for many years.

In a week his kidney difficulty disappeared, and by keeping up the use of simple water he has been well ever since —in that respect as well as he ever had been in his life. He said he told me these things because he thought it was his duty, as well as that it was due to me to know them.

He said that during the time he had the urinary trouble he drank but little water, did not seem to want it; had no thirst, and, therefore the fluids he did take consisted mostly of the tea and coffee drank at meals. He told me that he saw through his case after reading my paper, or at least he thought he did, and commenced drinking water in quantity sufficient to supply the actual wants of his body, and got well immediately; and by continuing to supply his body with the quantity of water it required, he has remained well ever since, so far as the urinary difficulty was concerned. But still more he was better in every way, and so far as I know has not needed the services of a physician since. And he was, when I last saw him in Zanesville, a very healthy and portly-looking gentleman. I think he has been absent, as I have not seen him for several monthes.

From his account no very definite conception can be obtained of the probable pathology of his case, other than that it was due to deficient water supply to his body. It may be definitely assumed that there was no organic lesions existing, or he would not have recovered at all, under any plan of management.

Several years since I found my professional friend and neighbor, Dr. Ball, of the city of Zanesville, in bed, with most of the prodromata of continued fever; hot; dry and uncomfortable. His usually clear head, thick and dull, eyes injected, sick stomach, pulse and temperature above natural. I proposed a watery saturation, to which he rather mechanically assented.

He thought a goblet of hot water was enough, but I thought otherwise, and induced him finally, to swallow perhaps seven goblets. But little came back by vomiting. From that hour the tide was turned, and his recovery was rapid, decisive and complete, without any other medication.

Only day before yesterday a gentleman from Barnesville consulted me, whose discomforts were mainly due to insufficient water supply of his body. He admitted that he never drank any water, as water, at all. Never took any fluids, except tea and coffee at meals, and of these combined, thinks he does not drink a pint in twenty-four hours. He passed very little urine, very high-colored. His food generally hurts him after eating, and he was seldom free from a burning sensation in the region of the stomach, and in front of the sternum. My prescription for his case was Bi-Carb. Potassa, in large watery dilution; several times a day, when the burning sensation was present, and an infusion of prickly ash bark after meals. I met him after dark two days later, and he told me he was much better in every respect.

I need not, however, multiply individual cases, though I could supply many illustrative cases of much personal discomfort, and actual suffering, from insufficient water supply.

A careful study of the uses and necessities of water in a living body can hardly fail to arrest attention. Thus, all food must be dissolved in water with the aid of acids, alkalies, or organic principles, as pepsin, etc. There can be no nutrition without this preliminary solution in water. Nor, on the other hand, can matter in effete conditions be conveyed out of the living body only as it is held in solution, or kept more or less soft, with water, as in the feces. The tendency of complex effete matter is to crystallization, in the absence of sufficient water to hold it in solution. Concentrated urine soon throws down what is called a sediment—that is, crystals of various chemical composition—salts of ammonia and lime. Stone in the bladder is an unmistakable instance of the crystallization of the effete matter in the body. What happens in the bladder in gross and palpable quantities, can certainly happen in the interstices of any of the soft tissues. It seems to me that this is the real pathology of the states known as "bilious," professionally, as well as popularly. There are often such cases which do not respond to what I call the "dry treatment," by pills and powders, which improve with wonderful rapidity at watering places, under thirty to forty tumblerfuls of weak saline solutions, natural mineral waters, per day. This is emphatically the "wet treatment." I have many reasons for thinking that for such cases the purer the water the better for the patient, provided the body is kept saturated day after day until all dead or effete matter is removed.

Consider again, if you please, the actual uses of water as a lubricator in living bodies. Each separate muscle, ligament, tendon, etc., requires to be lubricated, not alone in its bulk, but each fiber in muscle, each mesh in connective tissue, each special histological element, or structure, in viscuses, must be lubricated. And Nature's lubricating material in living bodies is mainly water, holding in solution some salts and albuminous compounds.

Four days since I visited a lady confined to bed, feet somewhat swollen, very painful when moved, complexion exceedingly sallow, no appetite, pulse not much accelerated, but small and thready, with a temperature barely half a degree above natural. I placed her feet in cloths wet with water, gave her small doses of calomel, $\frac{1}{2}$ grain, three times a day, and a large volume of saline solution, epsom salts, etc. Here was a living body, with nearly as much dead matter as living matter in it. The tissues of the extremities needed lubricating badly, and I could do it more effectually by external applications of water than by internal applications alone. In the continuous bath, the object was speedily accomplished, and to-day they are discontinued as being no longer necessary, and her general condition keeps pace with the improvement in her extremities. I apprehend a full recovery of her ordinarily fair complexion will occupy a month or more, though three-fourths of the time she will be able to go about more or less. Had she a steady temperature of 100 to 102°, her recovery would be more rapid. But her temperature remains altogether natural, so that her full recovery will be more tardy.

Consider once more that water is all the time escaping from living bodies, by the skin, from the lungs, and into and from the bladder. The quantity lost in other ways than through the bladder must amount to several pounds a day. Add to this the twenty to sixty ounces from the bladder each 24 hours, and the waste of water is seen to be very considerable.

Is it probable that each and every human being supplies this demand properly every day? I think not, for I find in actual practice many who do not, and suffer in many ways for their neglect.

When it is not duly supplied from the exterior, it has to be made in the interior of the body, from any available material, to make up, as far as possible, for the deficiency. It is probable that some portion of the deficit is supplied from the ingoing atmosphere at the lungs, as well as at the cutaneous surface; thus reversing the order of nature. Some is undoubtedly made during the retrograde metamorphosis of the tissues, as they are wasted in the performance of functional duty. But a still larger proportion is made from other available material in the body. Oxygen, introduced in the respiratory process, as well as in food, is made to combine with the hydro-carbon contents of the colon, or taken from whatever source it can be had, to form water.

The failure on the part of living beings, and I speak and have in my mind, as a central idea, human beings, but I think the general principles underlying apply equally to all organic life, animal and vegetable, to properly supply the wants of their bodies with water, entails burthens on their organic systems which are sure to be felt, sooner or later, at some local point, as a so-called disease. And generally in derangements of nutrition—a prominent feature of all so-called chronic disease—though no special form of ailment results, entitled, according to established professional usage, to be ranked as a distinct "disease." And it is no part of my present purpose to identify any so-called new disease. They are far too numerous already for the good of either physicians or patients. They do not exist in fact, or nature, but are the out-growths of scholarly refinement and classification—in a word, they only exist in the minds of patients or practitioners.

Defective water supply must contribute no little towards the modifications of structure, which underlie all so-called chronic diseases, and do something towards exaggerating the changed functions which proceed from them in consequence of changes of structures.

What has seemed to be most remarkable in those suffering from deficient water supply, is their unconsciousness of the cause of, at least a part of their discomfort. They say they do not want water, and therefore do not drink it. And I have found no little difficulty in getting them to drink water, as water, notwithstanding they admit they feel better for it.

In searching for the cause of this utter loss of the sense of thirst, I have been unable to reach any other conclusion than that it is due to a paralysis, so to speak, of the sense of thirst; or perhaps, more properly, sensation of thirst. The condition, it seems to me, requires a term to express it, I therefore propose to call it *Hydroadipsia*, absence of thirst for water; water thirst absent, paralyzed, lost. Adipsia means absence of thirst; but it does not express absence of thirst for any particular fluid. Thus, in my own person I have an absence of thirst for alcoholic beverages; have, in fact, as much aversion to them as some of my hydroadipsic patients have for water. It is, therefore, no cross or no merit for me not to be a dipsomaniac. It is to limit the term adipsia to absence of thirst for water that I prefix hydro to it; and the meaning of the term from its derivation, is exactly what I desire to express, absence of thirst for water.

Coffee and tea tippling has risen to the dignity of a first-class vice in most civilized countries—England and the United States being the largest consumers in the world— and contributes in no small degree to restrictions on the necessary water supply to those using them moderately. Limiting, as they do, the speed and extent of molecular transformations of material in living bodies, they necessarily, and unavoidably, lay the foundations for many minor derangements of structure and function.

In prescribing for such cases—cases of hydroadipsia—if they are in fair flesh, I find it best to give largely of weak saline solutions, as bi-carb. potassa, chloride of ammonium, citric acid, and sometimes lime water; or if badly nourished, weak solutions of muriatic acid, or elixir vitrol, with often small doses of calomel, half grain, three times a day, or every six hours, until the speed of molecular transformation is advanced; then weak infusions of prickly ash, black alder, poplar, or some other barks with peculiar bitter principles; sometimes combining with them valerian. The purpose is to keep up the water supply, rather than introduce "stored up force" in medicines, unless there exists special indications for them.

Most persons laboring under hydroadipsia—deficient water supply of their bodies—who follow my instructions for any length of time get better, and remain better until they allow the water supply to fall short again.

It must not be concluded, therefore, that chronic sufferers from hydroadipsia are permanently cured of their ailments by the means I have pointed out. The difficulty in keeping up a proper water supply is too great for confirmed hydroadipsics to overcome without continuous effort, and realizing that a proper water supply is an essential condition of personal comfort and health.

But too large an amount of good can be done in the way and by the means I have pointed out, to be neglected in the professional management of such cases.

In the management of acute complaints, particularly of fevers, the old traditions of limiting the water supply under some vague idea of its doing harm, particularly when the stomach is irritable, still lingers to a considerable extent among the people, and is by no means extinct even in the professional mind.

I attended in January last, a case of pneumonia, in which the water supply had been limited, though the patient urgently demanded it, because there was occasional sick stomach. This was prior to my having charge of the case. Almost my first proceeding was to give the patient a watery saturation, so to speak, by allowing him to drink what he thought satisfied him, then by coaxing getting nearly as much more swallowed; very little came back by vomiting, the want was cancelled, and the water supply was kept up by lemonade during the remainder of the time he was under professional notice.

I often give water as hot as it can be swallowed in large volume, say one to three pints, or more, at a time, to flood or saturate the system. In doing so it is better to make it palatable with common salt. It should be strictly boiling, and sipped from tablespoon or small ladle, blowing each portion with the mouth until it can be swallowed without scalding. As it is thus introduced very gradually, a very large amount can be taken without sick stomach, or sense of fullness. It is absorbed in the blood-vessel system, the volume and frequency of the pulse becoming more natural in a very marked degree, followed by a glow of warmth all over the cutaneous surface, which is, in many instances, speedily bathed in perspiration. The surplus not needed by the system soon escapes by the skin or through the kidneys.

Thus, three months since, I found a gentleman something over fifty years of age with pulse hardly countable, exceedingly thready, surface and extremities icy cold, intellect confused, complaining only of weakness and oppression in the chest, and as it seemed to me, in the utmost danger. He must be got warm, and his circulation must get fuller and slower before he could be considered safe. I knew of no safer and speedier way than flooding his system with hot water. But there was none in the house; and though it is said the " watched pot never boils," it did boil at length, and my patient began to sip from a tablespoon, protesting all the time that he did not want it, could not take it, and did occasionally refuse it. But I persisted, and got something over a quart swallowed; in the meantime having his feet in a warm bath. From being a mere thread at the commencement, the pulse had risen to a fair volume, and had fallen in frequency to near the natural standard; he felt better, was laid on a lounge and in a little while was asleep. When he awoke, two hours later, he was apparently altogether relieved, but took during the night three half-grain calomel granules, which moved his bowels next morning, and he resumed his usual business activity. In the evening he had a galvanic application, feet in hot water connected with carbon pole, zinc pole at back of neck, spine and chest.

This gentleman told me he drank very little water as a general thing, did not feel any want of it, relied mainly on tea or coffee at meals. My impression was, at the time, that he had brought about his condition by deficient water supply. I explained to him the necessity of more water, asked him to drink water as water. He has not been sick since.

With free heat and water—water at the highest temperature it can be swallowed—made palatable with salt, it seems to me I do a large amount of good in many apparently diverse conditions, though the central fact runs through all, of deficient water supply. I think I relieve more headaches in this way than by all other means combined.

It has the objection of consuming the physician's time in giving it, as patients left to themselves rarely ever take enough to do them the most good in the shortest time. The fact that life momentarily depends on molecular changes of matter, can not be explained to them so that they can understand it; and so fail to comprehend how it can do them good when they do not feel the want of it. I generally have to remain with my patient for whom I prescribe it, and give it in person.

The result of my experience, observations and inquiries on this subject may be stated as follows:

1. That a water supply, with the limits not at present well defined, is a necessary condition of healthy human life, probably depending on the extent of molecular work done; more work, more water; less work, less water; but not made known in many cases by the sensation of thirst.

2. That a large number of human beings seemingly have not, or if they ever had, have lost the sensation of thirst for water, and therefore fail to take the water supply required for the healthy performance of the various functions of their bodies, and constitute a class of true hydroadipsics.

3. That a prominent feature of professional investigation into the nature and causes of so-called chronic diseases, should, in each individual case, include inquiries into the water supply of their bodies; and if found deficient, make adequate provisions for supplying it, either by water as water, or weak saline solutions, or vegetable infusions.

4. That like defects in water supply in acute affections, especially continued fevers, frequently occur, and should receive professional attention.—*Lancet and Observer.*

